# Novel Insights into Molecular Mechanisms of Endometrial Diseases

**DOI:** 10.3390/biom13030499

**Published:** 2023-03-09

**Authors:** Tullio Golia D’Augè, Ilaria Cuccu, Giusi Santangelo, Ludovico Muzii, Andrea Giannini, Giorgio Bogani, Violante Di Donato

**Affiliations:** 1 Department of Maternal and Child Health and Urological Sciences, Sapienza University of Rome, Policlinico Umberto I, 00100 Rome, Italy; 2 Department of Medical and Surgical Sciences and Translational Medicine, PhD Course in “Translational Medicine and Oncology”, Sapienza University, 00185 Rome, Italy; 3 Gynecological Oncology Unit, Fondazione IRCCS Istituto Nazionale dei Tumori di Milano, 20133 Milano, Italy

Endometrial diseases are the most common gynecological pathologies in Western Countries. Among these, endometrial cancer (EC) is the most common gynecological malignancy [1], the fifth-most-diagnosed cancer in women worldwide and one of the leading causes of cancer mortality among women in developed countries [2]. In 2020, more than 417,000 new EC cases were estimated to be diagnosed worldwide. Its incidence has also risen due to the increase in risk factors in the female population, especially obesity and aging [3].

Endometrial hyperplasia is considered a precursor of endometrial carcinoma and according to the World Health Organization (WHO) classification of 2014, and it is classified in atypical hyperplasia or non-atypical hyperplasia [4]. In clinical practice, it is important to distinguish these two forms, because atypical hyperplasia is considered a premalignant condition that requires precise management. Due to changes in diagnostic criteria and methods, hormone therapies used in clinical practice and a possible concomitant carcinoma, it is difficult to obtain a reliable estimate of the true incidence of endometrial hyperplasia. In patients with atypical hyperplasia, there could be concomitant endometrial cancer in 32–37% of cases with a risk of progression of up to 25% [5,6,7]. At present, among 14% of patients with EC are women of child-bearing age and a large percentage of fertile women delay the age of first pregnancy, so the number of nulliparous women diagnosed with EC is increasing [8,9].

Thus, in recent years, numerous studies have been developed to refine the clinical management and personalization of patient therapy with EC, considering not only traditional prognostic factors but also an innovative molecular analysis with the aim of defining different classes of risk and developing therapies targeted to the molecules involved in carcinogenesis.

In 2013, the Cancer Genome Atlas (TCGA) Research Network changed the approach to EC classification with the integration of molecular characterization, resolving the numerous limitations in risk stratification that, for decades, were based only on tumor grade and histotype, depth of myometrial invasion and cervical and adnexal involvement. Based on mutations and somatic copy-number variations, genome and exome sequencing and microsatellite instability (MSI) assay, it is possible to divide EC into four groups, each with a different prognosis in term of specific progression-free survival and recurrence risk: Polymerase epsilon (POLE) ultramutated, MSI hypermutated, copy-number (CN) low and CN high [10]. The first group has an excellent prognosis with low risk of recurrence and less than 1% mortality. It commonly occurs in young women with low body mass indexes. This subgroup comprises EC low and high grade and is characterized by somatic mutations in the exonuclease domain of Polymerase epsilon DNA [3,11,12,13]. MSI hypermutated group has intermediate prognosis and it is caused by defects in DNA mismatch repair (MMR) systems. The molecular aberration is caused by MutL protein homolog 1 (MLH1) promoter hypermethylation that determines to silence one of the key genes of DNA mismatch repair (MMR) systems [3,12,14,15]. Copy-number (CN) low group, also called microsatellite stable, has tumor Protein 53 (TP53) wild type and POLE wild type and expresses high levels of estrogen and progesterone receptors (ER/PR). This subgroup includes most endometrioid tumors of low grade and considering that it has a low number of somatic alterations, usually its prognosis is good [3,16,17]. Copy-number (CN) high is the group with the worst prognosis, including 8–24% of EC. The genetic alterations most frequently present are P53 abnormalities and a high number of somatic alterations. Usually the EC CN-high are high-grade tumors and the most common tumors are serous and mixed carcinomas [3,16,17,18,19]. Moreover, a new model called ProMisE (Proactive Molecular Risk Classifier for Endometrial Cancer), based on the Institute of Medicine (IOM) guidelines, has been introduced to exceed the limits of the methodologies used for the TCGA study, such as cost, complexity and unsuitable for immediate clinical application. Numerous relevant studies demonstrated the validity of this model if applied not only to the final hysterectomy specimens but also to diagnostic specimens such as endometrial biopsies or curettages [20,21]. This model has been applied in the recent European Society of Gynaecological Oncology (ESGO)–European Society for Radiotherapy and Oncology (ESTRO)–European Society of Pathology (ESP) 2020 Guidelines for the management of EC patients according to the tumor aggressiveness and the likelihood of recurrence, with the objective to use molecular and genomic profiling with the histopathological features to determine the most appropriate and tailored adjuvant strategies [22]. Other genetic mutations were analyzed with the aim of refining the characterization of the four subgroups. As we all know, the p53-mut EC has the worst prognosis and data demonstrated that, in this subgroup of EC, L1 cell adhesion molecule (L1CAM) expression >10%, protein phosphatase 2 scaffold subunit alpha (PPP2R1a) and F-box and WD repeat domain containing 7 (FBXW7) mutations and histological grade 3, without hormone receptor expression were the prognostic factors that increased the risk of recurrence and a decrease in overall survival. MSI and no specific molecular profile (NSMP) group frequently explain AT-rich interaction domain 1A (ARID1a) abnormal expression and catenin beta 1 (CTNNB1) mutant, respectively. EC patients carrying CTNNB1 exon 3 mutations had an increased risk of distant recurrence. Molecular characteristics found in EC with worse prognosis are Estrogen receptor (ER) positivity, phosphatidylInositol 3-Kinase/protein-kinase B (PI3K/AKT) mutations, progesterone receptor (PR) positivity, L1CAM positivity. These results suggest that it would be useful to perform a more detailed molecular analysis especially in high-risk ECs, which represent a subclass with complex and heterogeneous characteristics, refining therapeutic management in clinical practice [21,23,24,25,26]. Furthermore, EC generally affects patients with a higher rate of comorbidity, elderly or obese, the assessment of the state of women’s frailty is fundamental to customize treatment strategies and reducing the morbidity rate therapy-related [27,28,29].

Considering the strong scientific evidence and the results we will obtain in ongoing studies, in recent years, EC therapy is increasingly becoming tailored for the various subclasses even if no level A evidence has supported the use of mutational and genomic profiling in the selection of adjuvant treatments in patients with early-stage disease. To date, only advanced or metastatic stages could benefit from targeted adjuvant therapies based on molecular alterations, particularly considering advanced MSI-H/MMR-deficient (dMMR), numerous studies have evaluated the efficacy of monoclonal antibody therapy directed against immune checkpoint-associated proteins, expressed at high levels within the tumor microenvironment and making tumor cells susceptible to immune system response [24].

However, molecular analysis could guide the therapeutic strategy for the treatment of precancerous EC lesions involving premenopausal women, nulliparous or with pregnancy plans, who would prefer conservative treatment. There are few data available in the literature that analyze how molecular classification might predict which subclasses have highest risk of evolution in EC. Results confirmed that, also in endometrial atypical hyperplasia POLEmut and CNL groups have a good prognosis compared to CNH and MSI-H groups [30] and Phosphatase and TENsin homolog (PTEN)-negative/b-catenin-positive combination expression could increase the risk of cancerous progression [31]. 

Unfortunately, few data are available for the use of molecular analysis for such diseases and improving risk classification for patients who may undergo fertility-sparing is one of the future targets. Endometrial diseases include a variety of pathologies that even within the same class may differ in histopathological or molecular features. Therefore, detecting and validating the use of molecular classification in precancerous lesions or analyzing different molecular markers could change therapeutic strategy, increasing the follow up of fertility-sparing patients or tailoring surgical radicality, reserving demolition surgery only for patients at high risk of cancer progression. 

More studies are needed to validate this scientific evidence that could revolutionize the clinical management of endometrial disease.
